# Green Management and Sustainable Performance of Small- and Medium-Sized Hospitality Businesses: Moderating the Role of an Employee’s Pro-Environmental Behaviour

**DOI:** 10.3390/ijerph20032244

**Published:** 2023-01-27

**Authors:** Ibrahim A. Elshaer, Alaa M. S. Azazz, Sameh Fayyad

**Affiliations:** 1 Department of Management, College of Business Administration, King Faisal University, Al-Ahsaa 380, Saudi Arabia; 2 Hotel Studies Department, Faculty of Tourism and Hotels, Suez Canal University, Ismailia 41522, Egypt; 3 Department of Tourism and Hospitality, Arts College, King Faisal University, Al-Ahsaa 380, Saudi Arabia; 4 Tourism Studies Department, Faculty of Tourism and Hotels, Suez Canal University, Ismailia 41522, Egypt

**Keywords:** hotel industry, green management, sustainable performance, pro-environmental behaviour, environmental performance, economic performance, social performance

## Abstract

As green management practices (GMPs) matter not only for improving the organizations’ tribble line performance (environmental, economic, and social) but also can sustain a competitive advantage. Since the tourism and hospitality industry is subject to environmental expectations from visitors, governments, and the community, it is vital to understand what motivates GMPs to overcome environmental obstacles and satisfy those demands. However, the current literature fails to comprehensively justify how small- and medium-sized businesses (SMEs) tackle green management difficulties when implementing their plans, even though these SMEs could be a leading contributor to environmental concerns. Although many scholars assert that employees’ pro-environmental behaviours are decisive in boosting efforts of green management to improve corporate sustainable performance, only limited studies probed the importance of employees’ pro-environmental behaviours in SMEs in developing countries. To fill this research gap, the data was gathered from 304 small- and medium-sized hotels and travel agency middle managers using a self-administered survey approach. The collected data was analysed using the Smart PLS-structural equation modelling technique. The PLS-SEM results demonstrated that GMPs can improve environmental, economic, and social performance and these relationships can be strengthened through the moderating effects of employees’ pro-environmental behaviour. The study findings revealed that small- and medium-sized hospitality businesses should focus on creating a culture of environmental stewardship and actively involve employees in green initiatives to enhance sustainable performance. The study is important as it helps to understand the role of employee pro-environmental behaviour in green management and sustainable performance in small- and medium-sized hospitality businesses and can help the industry to adopt more sustainable practices. Several theoretical and practical implications were discussed and opportunities for further research were elaborated.

## 1. Introduction

With the advent of the real environmental movement in the mid-1960s, which developed relatively rapidly over the next decades because of the continued use and waste of non-renewable resources, and the dramatic increase in the consumption rates, waste, and environmental pollution. Society began to blame corporations for many of the world’s environmental woes, and they were held accountable for finding solutions; firms had little option but to try to integrate green management practices into their operational processes [1,2]. Over time, green management became a famous slogan internationally in the 2000s, and managerial leaders discovered that business and environmental objectives should be the same [3]. While adopting eco-friendly conscious strategies enables organizations to hold their social responsibility and do what is morally right toward the environment [4,5,6], it also helps businesses at the same time to stay competitive in their markets, improve their financial outcomes, firm value, product innovation, and sustain them over time [7,8].

According to institutional theory and stakeholder theory, [9] businesses often engage in green practices and innovation to avoid economical costs and political pressure [10,11], and to satisfy various stakeholders’ expectations by complying with social and moral norms [12], as well as to overcome competitors’ mimetic pressure [13]. As a result, firms accepted the notion that effective green management has the power to meet the three sustainability principles, namely economic success, environmental integrity, and social equality [14,15]. Generally, green management practices attempt to improve a firm’s sustainable performance by converting inputs (natural materials and auxiliaries) into products or outputs (goods and services) by emphasizing the balance and synergy of economic, social, and environmental advantages [16].

Furthermore, workers’ realization of the significance and gravity of environmental concerns can satisfactorily respond to such problems by engaging in pro-environmental activities that reduce resource waste and save operating expenses [17]. Therefore, previous research found the importance of employees’ pro-environmental behaviours in boosting efforts of green management to improve corporate sustainable performance [18].

In developing economies, small- and medium-sized enterprises (SMEs) contribute up to 40% of the gross domestic product. These figures rise even higher when informal SMEs are included. At the same time, these SMEs around the world add 60–70% of the global pollution [19]. Although there is a critical influence of green management practices on sustainable corporate performance, namely “environmental performance, financial performance, and social performance,” few studies have explored this relationship, especially in small- and medium-sized hospitality businesses (e.g., hotels and travel agents) in the context of emerging markets, especially in developing countries [16]. Furthermore, many scholars have advocated for empirical research to explore employees’ pro-environmental behaviours at the workplace to investigate their role in going green strategies [20]. However, limited studies probed the importance of employees’ pro-environmental behaviours in developing countries, where issues and challenges linked to the environment are specifically salient to SMEs in emerging markets [17]. As a result, there is a recognised need in the hotel and travel agent sectors for further research that investigates the outcomes that green management may achieve at the triple bottom line (TBL): economic, environmental, and social performance, given that SMEs may face additional constraints due to a lack of knowledge and resources to invest in strategies of green management [21].

Seles et al. [22] argued that institutional theory is the most suitable fit to illustrate corporate sustainable performance within the social and environmental dimensions. Stakeholder theory is also one of the practical approaches in social, environmental, and sustainability management examination, providing a starting point for investigations in a massive number of publications on corporate sustainability and sustainability management [23]. In the same vein, Hart [24] proposed “a natural resource-based vision of the firm”, which advised enterprises to use three interconnected strategies to gain a competitive advantage: pollution avoidance, product stewardship, and sustainable performance. Accordingly, the originality of this study, according to the institutional theory, the stakeholder theory, and the natural-resource-based view, is to examine the relationships between green management practices and corporate sustainable performance, namely environmental, economic, and social performance in SMEs in the context of emerging markets in developing countries and the moderating effects of employees’ pro-environmental behaviour. In short, the current research tries to give answers to the following questions: what are the influences of GMPs on small- and medium-sized hotel and travel agencies’ sustainable performance? Furthermore, is there a connection between GMPs at small- and medium-sized hotels and travel agencies and sustainable performance (environmental, economic, and social) moderated by the employees’ pro-environmental behaviour? Thus, in the realm of green management research, this study strengthens the empirical evidence from developing countries. Green management practices are essential for achieving sustainable performance for the hotel and travel agents. By implementing policies and practices that support and encourage pro-environmental behaviour among employees, organizations can improve their environmental performance, reputation, and financial performance.

## 2. Theoretical Background and Hypotheses Evolution

### 2.1. Theoretical Background of the Study

In the context of the social sciences, Deegan et al. [25] indicated that it is always preferable to get profound insights through more than one theory to gain a more comprehensive grasp of the practice. Therefore, our study tries to construct an integrated theoretical framework for explaining green management practices (GMPs) by small- and medium-sized hotels and travel agencies by integrating two mainstream theories, i.e., institutional theory and stakeholder theory, which have been employed in the green management literature by considering theoretical predictive motivations of GMPs. According to Fernando and Lawrence [26], the two theories are connected and complementary rather than competing. Most significantly, they may be included and related to GMPs in order to justify the motivations for such practices from a multi-theoretical standpoint. Based on stakeholder theory, sustainability management requires enterprises to provide “an important contribution toward sustainable development of the economy, society, and the ecological environment [27]. Here, in response to stakeholder concerns, corporations include non-financial indicators in green CEO compensation, holding them accountable for their eco-friendly behaviour and, consequently, their influence on sustainable performance [28]. This is because, if a CEO operates in a stewardship capacity, protecting the corporation and the ecosystem, a corporation’s green practices and innovations will be improved [29]. Similarly, according to institutional theory, institutional pressure affects businesses to incorporate environmental and social matters into their corporate strategies, products, and services, leading to improvements in the sustainability performance [30]. Consequently, we can argue that, according to the institutional theory and stakeholder theory, managers of the small- and medium-sized hotel and travel agencies seek to respond to the pressures and motivations of stakeholders and the institutional pressures and motivations by adopting GMPs to improve sustainable performance (environmental, economic, and social), and at the same time, these pressures and motivations may help to develop employee’s pro-environmental behaviour which may support the relationship between GMPs and sustainable performance.

### 2.2. Green Management and Sustainable Performance

**Figure 1:**
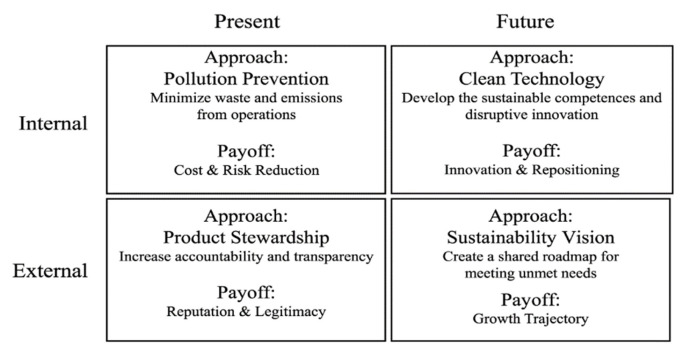
Hart’s (Hart [32]) strategy framework.

Green management is a type of environmentally conscious business management that concentrates on the voluntary prevention or continuing decrease of pollution, waste, and emissions [31]. Through the experimental examination of the literature handling historical, practical, and theoretical views, Pane Haden et al. [1] defined green management as “ the organization-wide process of applying innovation to achieve sustainability, waste reduction, social responsibility, and a competitive advantage via continuous learning and development and by embracing environmental goals and strategies that are fully integrated with the goals and strategies of the organization”. Accordingly, companies’ green management must go beyond legal issues and involve conceptual practices and tools such as green production, green marketing, green design, and incorporating green considerations into the organizations’ long-term goals [15,32,33]. Figure 1 shows Hart’s [32] strategy framework that describes how businesses could use the practices of green management to increase profitability by boosting corporate green sustainability.

Employing the natural resource-based view, businesses became confined by and dependent on ecosystems. In other words, strategy and competitive advantage will likely be based on qualities that enable ecologically friendly economic activity [32,33,34]. Therefore, corporate sustainable performance became the primary aim of the business and academic studies. According to the triple bottom line (TBL) approach, corporate sustainable performance is measured across three essential indicators: social, environmental, and economic [35,36]. Economic performance is evaluated in terms of operation and finance indicators. It is operationally related to organizations’ capacity to reduce input prices, energy consumption, and waste treatment and disposal [37]. Financially, it is measured by market share, profitability, and return on investment (ROI) [38]. Environmental performance is related to businesses’ capacity to conserve energy, reduce waste, and reduce the use of hazardous inputs [39,40]. While social performance evaluates the degree to which an organization contributes to society beyond economic interests, such that the industry generates a profit and its actions do not harm society [41].

However, some investigations have proven no link between green management and financial performance [42,43]. Handoko [44] found that green management positively influences financial performance, market performance, and society’s welfare. Specifically, green management positively influences financial and operational performance through reduction in production costs, minimized environmental damage, efficient energy consumption, minimized waste, adoption of recycling, raw material and water consumption saving, and potential open opportunities for green markets that have yet to be primarily recognized. Furthermore, enhancing the company’s image and green technology, improving the strategy the for firms’ competitiveness, and increasing social and health benefits [16,45,46] ultimately positively affects the economic performance of the firm. Consequently, we can hypothesize the following:

**Hypothesis** **1** **(H1):** Green management is positively associated with economic performance.

Adopting green management strategies can help a firm improve its environmental performance (EP) [47], by decreasing solid and water waste, carbon emissions, the usage of contaminated and harmful inputs, the commonness of environmental mishaps, and the general ecological effect of a firm’s operation [48]. Based on this discussion, the following are hypothesized:

**Hypothesis** **2** **(H2):** Green management are positively associated with environmental performance.

**Figure 2:**
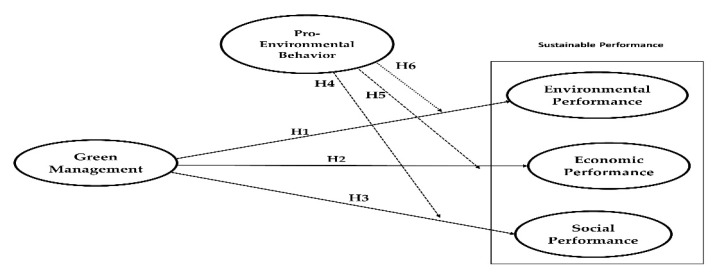
The research model. Dotted line: moderating effects; solid lines: direct effects.

Regarding social performance, adopting green management practices improves employees’ conditions as the local residents, allowing individuals to enjoy a healthier life [49]. Similarly, it is recognized that the most significant organizational benefits of tackling green management practices are increased social responsibility awareness among employees, as well as talent recruitment and retention [46]. There is also evidence that corporations that engaged in social responsibilities saw significant rewards in terms of customer and employee satisfaction, great personnel recruiting, and innovation, all of which are likely to strengthen a firm’s social performance [49,50]. These arguments direct to the following hypothesis, as illustrated in Figure 2:

**Hypothesis** **3** **(H3):** Green management is positively associated with social performance.

### 2.3. Employee’s Pro-Environmental Behaviour as a Moderator in the Relationship of Green Management and Sustainable Performance

Many businesses are content to meet only the minimal environmental legal obligations by adopting a reactive environmental strategy [51]. Because green management commonly does not create short-term profits and often takes a longer time, with increased spending of resources, to contribute to a business’s profitability [52]. In contrast, companies that voluntarily and discretionarily adopt green management practices tend to move beyond their legal obligations to participate in more effective environmental protection, i.e., adopting a proactive environmental strategy [53]. Therefore, green management requires employees to be inspired, empowered, and pro-environmental for greening to succeed [15]. Accordingly, an organization’s effectiveness in developing and enforcing multiple firm-level green programmes relies on its employees’ pro-environmental behaviours [54]. Employees’ pro-environmental behaviour is “discretionary/voluntary/non-voluntary acts that lead to [the] sustainability of organizational environment(s) which are not directly part of formal environmental management policies or systems” [55]. Employees whose pro-environmental behaviours can conserve resources by shutting off unneeded electrical equipment, utilising stairs instead of elevators, utilising double-sided papers for printing, and getting rid of unnecessary waste to save the natural environment [17,56]. Pro-environmental behaviours will not only contribute to the greening of firms but will also positively influence climate change and stop further environmental and ecological degradation [54]. Additionally, it directly contributes to the organisation’s financial and non-financial success [57]. Accordingly, this study suggests the following hypothesis:

**Hypothesis** **4** **(H4):** Pro-environmental behaviour moderates the influence of green management on environmental performance.

**Hypothesis** **5** **(H5):** Pro-environmental behaviour moderates the influence of green management on economic performance.

**Hypothesis** **6** **(H6):** Pro-environmental behaviour moderates the influence of green management on social performance.

## 3. Methods

### 3.1. Data Collection Process and Sampling Selection

This study explores the connections between green management practices and sustainable performance with the moderating role of employees’ pro-environmental behaviour. The study was directed and conducted on employees at the managerial level at SMEs (e.g., hotels and travel agents). Hotels and travel agents were selected as the targeted sample, as these two industries are closely related and have a significant impact on the environment. By including both industries in the sample, the study aims to examine the relationship between green management and sustainable performance in these two industries and how employee pro-environmental behaviour may moderate this relationship. A drop-and-collect method was adopted depending on the research team personnel connections and relationships. The research team are engaged in the hospitality sector, as they are academics in the tourism and hotel management faculty and have wide connections with human resource managers in the field. The survey was delivered to HRM in hotel and travel agents to redistribute it to employees in the middle-managerial levels to complete the survey. Employees in the middle managerial level were selected as they are considered the linking chain between normal employees and the top management and have the ability to provide the required data. The employed scales were adopted from English literature. Consequently, we translated the scales into Arabic so that the targeted employees could entirely understand. We employed a convenience sampling technique due to its time- and money-saving merits in contacting respondents [8]. A total of 304 (170 from hotels and 134 from travel agents) valid replies were obtained after dropping the questionnaires in October and November 2022. The sample size of 304 responses in the current study is appropriate for analysis using PLS-SEM, as it meets Nunnally’s [58] recommendation of at least 10 responses per scale item (the study has 26 scale items, for a minimum recommended sample size of 260) and conforms with the criteria of Hair et al. [59] for at least 100–150 responses to generate adequate estimations.

Because respondents’ privacy is a substantial issue, we begin the questionnaire introduction with a statement to explain the study’s primary purpose and the strict confidentiality that would be preserved with any data collected. The researchers used the independent sample t-test to compare the mean scores for early and late responses and found no significant differences (*p* > 0.05), indicating that non-response bias is not a concern in this study [60]. Furthermore, the researchers used Harman’s single factor procedure and SPSS v21 to test for the presence of common method variance (CMV) by analysing all study variables through exploratory factor analysis (EFA) without rotating the factors. As a result, they found that one dimension was able to explain 33% of the variance, indicating that CMV is not an issue in this study. The previous results were confirmed by checking the VIF values, where no value was found to exceed 5, which further confirms that CMV is not an issue in our study. 

### 3.2. The Study Measurements 

All the measures employed were obtained from previous measures that showed adequate psychometric properties and were interrelated to our study theme. All factors, with their reflective variables and references, are shown in Table 1. 

### 3.3. Data Analysis Techniques 

“Structural Equation Modelling” (SEM) and “Partial least squares” (PLS) were employed so that the justified relationships with moderating effects of employees’ pro-environmental behaviour could be explored and evaluated. PLS-SEM allows the retention of a larger number of reflective items per factor than other statistical techniques. We followed Leguina’s [61] two-step approach in analysing the collected data. In this approach, the measurement model should first be evaluated for reliability and validity, and then the structural model can be assessed for hypotheses testing and confirmation. To evaluate the measurement model, we adopted the suggested criteria introduced by Hair et al. [62], which contain several threshold metrics such as the “standardized factor loading” (>0.7), the “composite reliability, CR” (>0.7), the “average variance extracted, AVE” (>0.5), the “normed fit index” (higher than 0.90), the “Standardized root mean square Residual, SRMR” (<0.08), the R2 (>0.1), and the Stone–Geisser Q2 (>0.0). 

## 4. The Study Results 

### 4.1. Descriptive Results

According to the results, 75% of the survey participants at the middle management level were male and 25% were female. In terms of age, 5% were over 20 years old, 15% were between 26 and 35, 35% were between 36 and 45, and 45% were 46 or older. Education levels showed that 20% had a secondary school certificate, 65% had an undergraduate degree, and 15% had a postgraduate degree. Additionally, 60% of the respondents worked in 5-star hotels, while 40% worked in the travel agent industry. Experience levels revealed that 15% had one year of experience, 30% had 2 to 4 years, 30% had 5 to 7 years, and 25% had 8 years or more.

### 4.2. The Outer Model Assessment (Measurement Model)

As proposed by Hair et al. [63], we have assessed for factor loadings, construct validity, reliability, internal consistency, averaged variance extracted (AVE) (Table 1), and dimensions discriminant validity with factor cross loadings (Table 2), Heterotrait–Monotrait Criterion (Table 3), and Fornell–Larcker Criterion (Table 4). As all the recommended minimum and/or maximum levels were adequate [63,64], the suggested outer measurement model is appropriate, and the scale shows good convergent validity [65].

Concerning the evaluation of dimensions discriminant validity, both the Fornell–Larcker and Heterotrait–Monotrait values complied with the recommended thresholds [66]. Therefore, the scale exhibited adequate discriminant validity.

**Table 1:** Psychometric Properties.

	Fac. Loadings	*a* Value	C.R	AVE
Recommended threshold	>0.7	**>0.7**	**>0.7**	**>0.5**
Economic performance (Econ_P) [67,68,69,70,71]		0.805	0.850	0.628
Improved market share	0.874			
Improved company image (i.e., company is seen as a green company)	0.806			
Improvement in company’s position in the marketplace	0.752			
Increase in profitability	0.729			
Environmental performance (Envir_P) [72,73,74,75,76,77,78,79,80,81]		0.913	0.927	0.659
Reduction of CO_2_ emissions	0.875			
Reduction of wastewater	0.863			
Reduction of solid wastes	0.732			
Reduction of energy consumption	0.896			
Decrease in production of toxic / harmful / hazardous / flammable substances	0.784			
Decrease in material usage	0.760			
Improved compliance with environmental standards	0.875			
Green management (Green_M) [82,83,84,85]		0.946	0.947	0.786
The management of my organization is highly committed to following environment-friendly policies.	0.891			
We regularly review and redesign our strategies to ensure their compliance with environmental criteria.	0.895			
Our organization is open to adopting new or improving existing management systems with respect to policies and practices.	0.893			
The management ensures the availability of infrastructure to improve the operational processes.	0.911			
Our management ensures that our production and service activities are environment-friendly.	0.895			
The management of our organization takes initiatives to raise awareness about the environmental issues and impacts of business operations.	0.833			
Pro-Environmental Behaviour (Pro_Envir) [86,87,88]		0.940	0.946	0.770
Performance appraisal records environmental performance concerns and policy	0.883			
Performance appraisal includes environmental incidents and responsibilities	0.876			
Employee gets reward for environmental management	0.875			
Employees are involved in becoming environmentally friendly	0.886			
Uses teamwork for resolving environmental issues	0.887			
Employees discuss environmental issues in team meetings	0.858			
Social performance (Soci_P) [53,81,89,90,91,92]		0.836	0.840	0.752
Improved relationship with the community and stakeholders	0.852			
Improved work safety	0.889			
Improved living quality of the surrounding community	0.860			

**Table 2:** Fac. Cross-loadings.

	1	2	3	4	5
1-Economical performance					
Econ_P_1	**0.874**	0.638	0.269	0.298	0.513
Econ_P_2	**0.806**	0.456	0.210	0.175	0.308
Econ_P_3	**0.752**	0.389	0.152	0.145	0.304
Econ_P_4	**0.729**	0.376	0.213	0.184	0.376
2-Environmental performance					
Envir_P_1	0.567	**0.755**	0.397	0.312	0.566
Envir_P_2	0.529	**0.875**	0.358	0.347	0.641
Envir_P_3	0.488	**0.863**	0.350	0.317	0.620
Envir_P_4	0.490	**0.732**	0.211	0.246	0.495
Envir_P_5	0.496	**0.896**	0.380	0.358	0.658
Envir_P_6	0.454	**0.784**	0.250	0.257	0.498
Envir_P_7	0.413	**0.760**	0.249	0.257	0.480
3-Green management					
Green_M_1	0.222	0.342	**0.891**	0.482	0.406
Green_M_2	0.226	0.349	**0.895**	0.476	0.409
Green_M_3	0.260	0.353	**0.893**	0.502	0.406
Green_M_4	0.279	0.388	**0.911**	0.541	0.452
Green_M_5	0.238	0.300	**0.895**	0.542	0.386
Green_M_6	0.235	0.380	**0.833**	0.612	0.427
4-Pro-Environmental Behaviour					
Pro_Envir_1	0.240	0.338	0.503	**0.883**	0.399
Pro_Envir_2	0.255	0.388	0.470	**0.876**	0.360
Pro_Envir_3	0.196	0.265	0.487	**0.875**	0.331
Pro_Envir_4	0.266	0.370	0.561	**0.886**	0.399
Pro_Envir_5	0.248	0.358	0.524	**0.887**	0.399
Pro_Envir_6	0.178	0.218	0.596	**0.858**	0.358
5-Social performance					
Soci_P_1	0.366	0.493	0.469	0.378	**0.852**
Soci_P_2	0.458	0.654	0.368	0.368	**0.889**
Soci_P_3	0.464	0.708	0.371	0.366	**0.860**

Bold items: “for discriminant validity, the outer factor loading of the reflective items have should have higher value than the cross-loading related scale measures”.

**Table 3:** HTMT Matrix.

Factors	1	2	3	4	5
1-Economical performance					
2-Environmental performance	0.681				
3-Green management	0.303	0.414			
4-Pro-Environmental Behaviour	0.285	0.390	0.631		
5-Social performance	0.580	0.806	0.520	0.479	

HTMT: Heterotrait–Monotrait ratio.

For proper discriminant validity, all HTMT should be less than the value of 0.90.

**Table 4:** Fornell–Larcker criterion matrix.

Factors	1	2	3	4	5
1-Economical performance	**0.792**				
2-Environmental performance	0.608	**0.812**			
3-Green management	0.275	0.399	**0.887**		
4-Pro-Environmental Behaviour	0.267	0.375	0.594	**0.878**	
5-Social performance	0.492	0.706	0.469	0.429	**0.867**

Bold values: “for a proper discriminant validity, AVE values (bold) have to show values that are higher than the inter-variable correlation coefficient”.

Following the suggestions of Sarstedt et al. [59], assessment of the outer model’s collinearity was needed in the next phase of the analysis process. Considering this, we calculated the VIF values for each reflective item, which should be less than 10 [62]; the findings demonstrated that all variables have VIF values less than 10. Hence, the data did not deteriorate from the multicollinearity problem. A bootstrap analysis technique was then conducted to examine the proposed hypotheses and their related t-values and significant *p*-values.

### 4.3. The Evaluation of the Inner Structural Model (Hypotheses Testing)

PLS-SEM was used to check the inner model for hypotheses testing once the outer model had been tested and confirmed to be accurate. The GoF of the inner model was evaluated by employing several criteria adopted from those suggested by [62,65,93]. Table 5 demonstrates that the model fulfilled all the conditions needed to approve its fit and prediction capability. The SRMR, R2, Q2, and NFI values exceeded the thresholds, permitting us further to assess the proposed hypotheses of the current study. 

Smart PLS4 has been conducted to run a bootstrapping method, which calculates the regression weights (β), t-statistics, and significance P level of the direct relationships and moderating impacts. As depicted in Table 5, we evaluated a total of nine hypotheses, three direct hypotheses, and three moderating effects.

**Figure 3:**
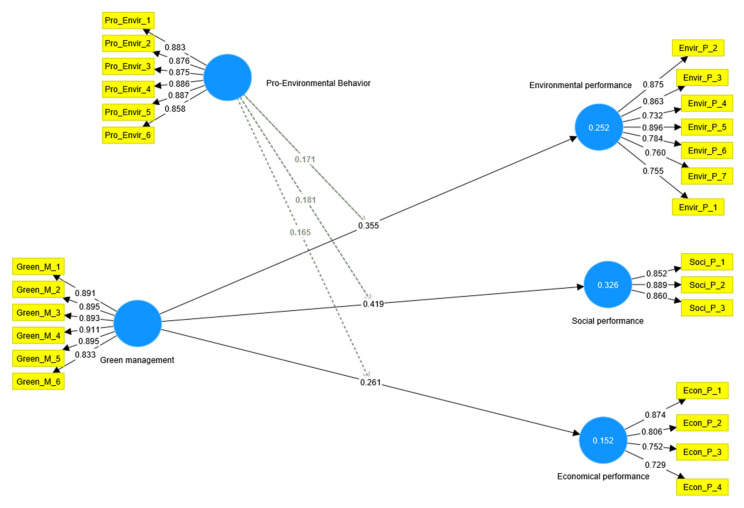
The study’s Inner and Outer model.

The findings, as pictured in Figure 3 and depicted in Table 5, demonstrated that green management had a significant (*p* < 0.001) and positive influence on economic performance (β = 0.261, *t* = 3.566, and *p* = 0.000), environmental performance (β = 0.355, *t* = 4.456, and *p* = 0.000), and social performance (β = 0.419, *t* = 6.997, and *p* = 0.000); consequently, H1, H2, and H3 can be supported. 

**Figure 4:**
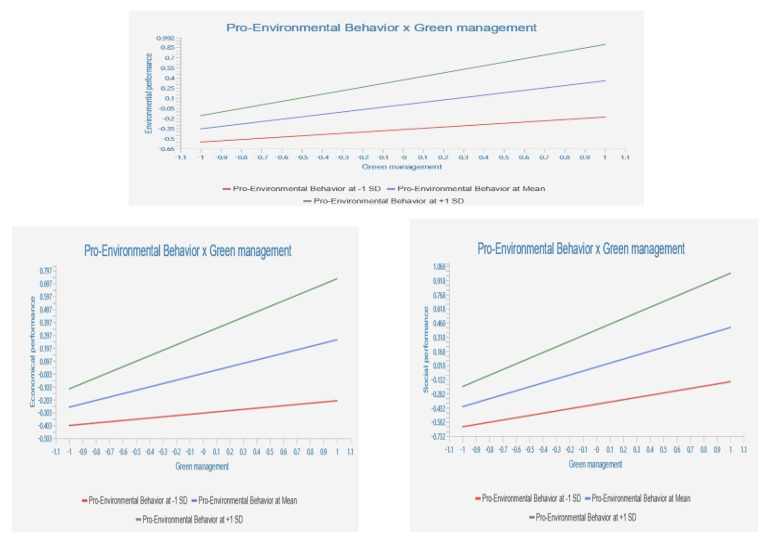
Simple Slope analysis for the moderating effects.

For the moderating evaluation, as pictured in Figure 4, the simple slope analysis approved the moderating effects of employees’ pro-environmental behaviour on the tested relationship. In more details, the Smart-PLS findings demonstrated that employees’ pro-environmental behaviour significantly improved the significant impact of green management on economic performance (β = 0.171, *t* = 2.600, and *p* = 0.010), which means that the result can support H4. Similarly, employees’ pro-environmental behaviour significantly improved the significant impact of green management on environmental performance (β = 0.171, *t* = 2.472, and *p* = 0.016) and social performance (β = 0.181, *t* = 2.649, and *p* = 0.008), permitting us to support H5 and H6. 

## 5. Discussion and Implication 

Although SMEs, including those in the hospitality industry, are critical to the contemporary economy—they represent over 80 per cent of all global enterprises [15,94]. However, their potential contribution to environmental problems is extensive. According to some estimates, they may be accountable for as much as 60 to 70% of carbon dioxide emissions and commercial waste [95,96]. Global competition, on the other hand, forces SMEs to enhance their organisational structures to apply environmentally friendly practise norms and standards [97]. This is contrary to some scholars who argue that the benefits of applying green management practices are relevant to larger companies only, not to SMEs [98,99]. Our study’s empirical results indicated that green management positively affects ECP, ENP, and SOP in small and medium hotels and travel agents. The results of the study show that the application of green management practices to SMEs produced a good impact not only to the environmental performance but also on economic and social performance, i.e., environment protection, cost savings, and improvement of employees’ and the local community’s conditions; all three have been proven to contribute to enhance the competitiveness of hotel and travel agent SMEs through product uniqueness, market expansion opportunities, and promoted image. According to these findings, small- and medium-sized hospitality businesses have switched from reactively addressing environmental issues to a proactive method to take advantage of the benefits of greening. In this context, Lee [100] indicated that SMEs are moving from a command-and-control technique to a market and competition approach in executing green management. SMEs, such as hotels and travel agents, can have advantages over large enterprises regarding guaranteeing effective green management: lines of communication are often shorter in smaller businesses, organisational structures are less complicated, employees continually fulfil numerous roles, and access to top management is more accessible. These features might be significant advantages for SMEs in terms of good green management [15].

Regarding assessing the moderating effect, studies of green management concerning SMEs are very scarce in the literature on business and management. Therefore, the main aim of the current research was to test the moderating role of employees’ pro-environmental behaviour in the relationship between green management practices and sustainable performance, i.e., environmental, economic, and social performance, in trying to understand how small- and medium-sized hospitality businesses adopt green management. The empirical results validated the positive moderation effects of the employees’ pro-environmental behaviour variable on the relationship between green management practices and environmental, economic, and social performance. In other words, according to the simple slope analysis for the moderating effects in Figure 4, employees’ pro-environmental behaviour strengthened the connection between green management and environmental, economic, and social performance. Accordingly, adopting green management practices will enhance the firm’s sustainable performance through employees’ pro-environmental behaviour at the workplace [101], while its absence would worsen environmental effects and weaken green management outcomes (e.g., environmental, economic, and social performance) [102]. Many scholars have also asserted that employees’ pro-environmental behaviours are critical for supporting environmental protection, boosting a corporation’s sustainable performance, and promoting the emphasis on eco-friendly activities like recycling, green initiatives, green engagement, and eco-supportive activities [103,104,105,106]. Accordingly, we can confirm that employees’ pro-environmental behaviour may help businesses overcome some obstacles to green management practices, such as a lack of resources and knowledge, high implementation costs, and a lack of long-term environmental goals.

## 6. Conclusions

On the basis of merging the relevant literature and theory, this article conducted an in-depth investigation of the association between green management practices and SMEs’ sustainable performance with testing the moderator role of the employees’ pro-environmental behaviour in this relationship and putting forward the corresponding study hypotheses. A total of 304 (170 from hotels and 134 from travel agents) valid datapoints were collected by means of questionnaires. Convergent and discriminant validity and the research hypotheses were evaluated using SEM with the Smart PLS program V.4. The findings approved that the scale has good validity. Furthermore, the findings showed that green management positively affects environmental, economic, and social performance (i.e., sustainable performance). The results also validated the positive moderation effects of the employees’ pro-environmental behaviour variable on the relationship between green management and the three sustainable performance components.

According to Freeman [107], in the framework of stakeholder theory, managers must understand the requirements and concerns of stakeholder groups in order to secure the necessary support for the corporation’s future survival. Similarly, institutional theory is established on the assumption that corporation options are not merely rational economic decisions but are also profoundly affected by external norms, values, and conventions [108]. Thus, businesses strive to adopt initiatives and practices to gain legitimacy or acceptance within the community to guarantee access to essential and scarce resources [109]. According to this theoretical lens, our research contributes to numerous paths in terms of theoretical and practical implications; the current study used the institutional theory, stakeholder theory and the natural-resource-based view to prove that green management practices enhance the triple bottom line (TBL): economic, environmental, and social performance with using the employees’ pro-environmental behaviour variable as a moderator between them in small- and medium-sized hospitality businesses (i.e., hotels and travel agents) in the context of emerging markets, especially in developing countries. Thus, the study responds to demands to explore the green management practices of SMEs in many countries, with the goal of shedding light on the similarities and variations [97,110], in addition to calls for empirical studies to investigate employees’ pro-environmental behaviours at the workplace to investigate their role in green strategy success [20]. As a result, the findings revealed in our study add to green management and behaviour theory and practice, as well as the SME literature. Finally, the study recommends supporting the employees’ pro-environmental behaviour to stimulate them to cope with environmental issues through task coping styles [111] to raise their expectations regarding sustainable performance [112]. Thus, this would overcome the obstacles of greening in small- and medium-sized hospitality businesses.

Similar to prior research on this area, the present study contains a number of limitations, and it is proposed that other research routes be pursued. First, the study tested the impact of green management practices on SMEs’ sustainable performance, while the employees’ pro-environmental behaviour role was examined as moderator; however, other variables can be explored and evaluated as moderators, such as employees’ experience, satisfaction, and/or loyalty, while different factors can be tested. Second, using cross-sectional data makes it impossible to determine the exact causal relationships between latent variables. In the future, researchers may use either numerous or longitudinal data sources to validate the structural model presented in this paper in a different context. Finally, the study only focused on small- and medium-sized hospitality businesses (hotels and travel agents), and the findings may not be generalizable to larger organizations or other industries.

## Figures and Tables

**Table 5:** The findings of the inner model.

Proposed Hypotheses	β	T-Value	*p*-Values	Results
Direct Paths				
H1- Green management -> Economical performance	0.261	3.566	0.000	Confirmed
H2- Green management -> Environmental performance	0.355	4.456	0.000	Confirmed
H3- Green management -> Social performance	0.419	6.997	0.000	
Moderating Effects				
H7- Pro-environmental behaviour x Green management -> Economical performance	0.165	2.600	0.010	Confirmed
H8-Pro-environmental behaviour x Green management -> Environmental performance	0.171	2.427	0.016	Confirmed
H9-Pro-environmental behaviour x Green management -> Social performance	0.181	2.649	0.008	Confirmed

GoF: Environmental performance, Social performance, and Economic performance R2 values exceeded the recommended cut-off point of 0.10. Environmental performance (Q2 = 0.182), Social performance Q2 = 0.272), and Economical performance (Q2 = 0.095) Q2 values exceeded the suggested threshold value of 0.0, SRMR = 0.042 (below the threshold of 0.05); and NFI = 0.967 (more than the cut-off value of 0.90).

## Data Availability

Data is available upon request from researchers who meet the eligibility criteria. Kindly contact the first author privately through e-mail.
